# Influence of Specimen Size and Test-Opening Geometry on the Sound Reduction Index Measured in Small-Scale Coupled Reverberation Rooms

**DOI:** 10.3390/s26134083

**Published:** 2026-06-27

**Authors:** Agata Polaczek, Katarzyna Baruch-Mazur, Dorota Młynarczyk

**Affiliations:** 1 Faculty of Civil Engineering, Cracow University of Technology, 31-155 Cracow, Poland; katarzyna.baruch@pk.edu.pl (K.B.-M.); dorota.mlynarczyk@agh.edu.pl (D.M.); 2 Agata Polaczek SZA Pracownia Akustyczna, 30-148 Cracow, Poland; 3 Andrzej Kłosak archAKUSTIK, 31-143 Cracow, Poland; 4 Department of Mechanics and Vibroacustics, Faculty of Mechanical Engineering and Robotics, AGH University of Krakow, 30-059 Cracow, Poland

**Keywords:** sound reduction index, airborne sound insulation, reduced-size specimens, test opening geometry, small-scale coupled reverberation rooms, measurement repeatability

## Abstract

The sound reduction index *R* is commonly determined using standardized laboratory procedures developed primarily for full-size building elements. However, in many research and development applications, including technical enclosures, lightweight panels, modular components, and new acoustic materials, only reduced-size specimens are available. In such cases, the influence of specimen dimensions and test-opening geometry on the measured sound insulation is not yet fully understood. This study investigates the effect of specimen size and geometry on the measured sound reduction index using a dedicated small-scale coupled reverberation room stand. Measurements were performed for five materials with different mechanical and structural properties: steel, polymethyl methacrylate (PMMA), medium-density fiberboard (MDF), gypsum board, and Sylomer. Six test openings were analyzed, including three square openings, one quasi-square opening, and two rectangular openings. The results show that specimen dimensions can significantly affect the measured values of *R*, especially in the low-frequency range, where modal behavior, boundary conditions, and the relationship between specimen dimensions and acoustic wavelength are important. The influence of specimen size was material-dependent and was more pronounced for stiff plate-like materials than for the highly compliant Sylomer specimen. Comparisons between square and rectangular openings with similar surface areas suggest that, within the investigated range of materials, specimen geometries, and measurement conditions, specimen surface area had a greater influence on *R* than specimen shape, although geometry can still contribute to the measured differences. The repeatability analysis confirmed that the measurement stand is sensitive to differences related to material type, specimen dimensions, and installation conditions. The proposed methodology may be particularly useful for comparative studies of novel acoustic materials and prototype building elements when only reduced-size specimens are available during the early stages of material development. The results support the use of small coupled reverberation rooms for comparative testing and preliminary material screening, while also showing that reduced-size sound insulation measurements require careful interpretation and cannot be treated as direct substitutes for full-scale standardized tests.

## 1. Introduction

The sound reduction index *R* is one of the fundamental parameters used to describe the ability of a partition to limit the transmission of airborne sound. The acoustic insulation of building elements is typically tested according to standardized measurement procedures defined in international standards. In building applications, the most commonly used set of documents is the ISO 10140 series, which covers laboratory methods for measuring the sound insulation of building elements [1,2,3,4,5]. These standards define the requirements for test facilities, measurement conditions, and procedures for evaluating airborne and impact sound insulation.

However, standard measurement methods have certain limitations, including those related to the test specimen itself. In laboratory tests of typical building partitions, the specimen should have an area of approximately $10\;m^{2}$, which is intended to ensure representative boundary conditions and realistic sound transmission paths [5]. Exceptions are made for small building elements, such as windows, doors, glazing, shutters, joints, or small technical elements. In such cases, the specimens may have smaller dimensions corresponding to the actual dimensions of the element or to dimensions specified in the relevant standardized procedures [1,2,5].

The ISO 10140 series is strongly rooted in the context of building acoustics and was developed primarily for elements used in construction. However, a problem arises when there is a need to evaluate the sound insulation of materials or systems that are not classical building elements. This applies, for example, to machine enclosures, panels used in vehicles, modular components, lightweight technical covers, or new materials developed in the research and development stage. Such applications often involve a different element scale, a different structural configuration, different boundary conditions, and a different installation method than those used for typical building partitions. At present, there is no widely accepted procedure that clearly defines how such small or non-building specimens should be prepared, installed, and interpreted in sound insulation measurements.

For this reason, selected research centers and laboratories have attempted to perform sound insulation measurements using specimens smaller than those required in standardized full-scale tests. Mleczko [6] analyzed airborne sound insulation tests of reduced-size partitions, indicating that the size of the specimen and the test opening is important when interpreting sound transmission loss results obtained for elements smaller than typical standardized specimens. Such an approach may be justified in comparative studies, preliminary material screening, and the design stage of new solutions, particularly when the available amount of material is limited or when preparing a full-scale specimen would be economically or logistically unjustified. For example, Balmori et al. [7] used a custom-made reduced-size transmission chamber for the comparative assessment of lightweight timber frame systems with recycled waste tire rubber layers, and then tested selected configurations in an accredited laboratory. A similar motivation can be found in studies on simplified and low-cost measurement setups, such as portable sound reduction boxes, developed for preliminary assessment of the insulation properties of materials without the need for expensive laboratory infrastructure [8]. A recent study by Del Rey et al. [9] also used a small transmission chamber to evaluate the sound reduction index of lightweight acoustic prototypes made from recycled plastic caps and sustainable materials, demonstrating the practical relevance of reduced-size setups for early-stage acoustic characterization of non-standard material solutions. Similarly, Vivolo et al. [10] used a small cabin (Soundbox) approach in the vibro-acoustic characterization of lightweight panels and reported the sound insertion loss (IL), defined as the change in the sound power radiated by a given source with and without the test object.

The use of smaller specimens can reduce material costs, simplify transportation, shorten installation and removal times, and make it possible to test a larger number of material or geometric variants. At the same time, reducing the specimen dimensions is not neutral from a physical point of view. This raises the question of whether and under what conditions a measurement result obtained for a reduced-size specimen can be considered representative of a larger element. Wareing et al. [11] experimentally investigated this problem and showed that both specimen area and selected construction parameters can affect the measured sound transmission loss. The dependence of the result on the finite dimensions of the specimen is also supported by theoretical models that describe the acoustic radiation of plates: Davy [12] analyzed the radiation efficiency of finite-size flat panels excited by incident sound, while Villot et al. [13] presented an approach to predict the acoustic radiation of finite-size multilayer structures based on models of infinite structures. In a related context, Hall et al. [14] showed that the structural layout of periodic Mindlin plates with non-uniformly spaced mass attachments can modify sound transmission loss in the coincidence region, further confirming the role of wave-related and geometry-dependent effects in plate transmission.

Theoretical models and classical works in structural acoustics and sound insulation indicate that sound transmission through a plate is not determined solely by its surface mass. Cremer et al. [15] describe in detail the propagation of bending waves in plates, resonant vibrations of damped systems, mechanical impedance, and the radiation efficiency of vibrating structures, thereby indicating that the acoustic response of a plate depends on its dynamic properties, damping, and the way in which structural vibrations are transformed into acoustic radiation. Fahy and Gardonio [16], in turn, analyze sound radiation from vibrating structures, sound transmission through partitions, and the coupling between the acoustic field and the structural response, which shows that the sound insulation of a panel depends on the combined influence of material parameters, geometry, excitation, and boundary conditions. With respect to the sound insulation of building partitions, Hopkins [17] emphasizes that the practical interpretation of measurement results requires combining experimental data with statistical and analytical models, because individual models have limitations resulting from their assumptions, and no single universal approach describes all sound transmission mechanisms over a broad frequency range. From the perspective of the present study, this means that when specimen dimensions are reduced, modal effects, edge support, mounting conditions, and the relationship between specimen dimensions and acoustic wavelength may become particularly important. These effects may be especially significant in the low-frequency range and near resonance frequencies or phenomena related to coincidence. It can therefore be expected that the influence of specimen dimensions on the measurement result will depend on the mechanical properties of the tested material, such as stiffness, density, thickness, and internal damping.

Previous studies have shown that the influence of specimen dimensions on measured sound insulation may be significant; however, the observed relationships are not always unambiguous and may depend on the type of system tested, the measurement setup, and the installation conditions. Wareing et al. [11] analyzed variations in transmission loss results caused by changes in specimen size and construction parameters and showed that changing the specimen dimensions can affect the obtained results, especially below the critical frequency. Other studies point to the significant role of specimen installation and support conditions in laboratory sound insulation measurements, especially for thin plates and small specimens, whose dynamic behavior may strongly depend on the mounting method [18].

In this context, the repeatability and reproducibility of sound insulation measurements are also important issues. Dijckmans and Vermeir [19] used a wave-based model to numerically analyze the variability of laboratory sound transmission loss measurements, indicating that repeatability depends, among other factors, on the measurement procedure, the number and positions of microphone points, and the position of the sound source. The authors also analyzed the reproducibility of results between different test facilities, demonstrating the importance of geometric parameters such as room dimensions, plate dimensions, and the configuration of the test opening. In a later study, Dijckmans et al. [20] extended this analysis to laboratory measurement procedures used in building acoustics, including the pressure method according to ISO 10140-2 and intensity methods according to ISO 15186-1 and ISO 15186-3 [21,22] in the low-frequency range of 50–200Hz. This results confirmed that the repeatability of measurement procedures depends on the positions of the source and receiving points, whereas the reproducibility of results between different facilities may be related to geometric parameters such as room dimensions, partition dimensions, the position of the test opening, and its thickness. The importance of this problem is also confirmed by more recent interlaboratory studies in which the scatter of sound insulation measurement results for a steel plate was analyzed across different laboratories [23].

In the case of small or non-standard specimens, these factors may become even more important, because even small differences in installation, pressure, or sealing of the specimen can lead to measurable changes in the value of the sound reduction index *R*. Therefore, the use of reduced-size specimens requires particular control of the measurement procedure and an assessment of the repeatability of the obtained results.

An alternative to full-scale reverberation rooms is the use of scaled measurement setups, which make it possible to conduct comparative studies at lower specimen preparation costs and with shorter installation times. Szeląg et al. [24] indicated that full-scale sound insulation measurements require extensive research infrastructure, a large amount of material, and the preparation of specimens with substantial dimensions, which increases costs, complicates transportation and storage, and limits the number of variants that can be tested. Scaled setups can reduce these problems, provided that appropriate geometric and frequency similarity relationships are maintained and the acoustic field conditions in the rooms are controlled [24,25].

Regarding the setup used in the present study, Szeląg et al. [24] described and validated coupled reverberation rooms at a 1:8 scale, representing a geometric model of the full-scale reverberation rooms at AGH University of Krakow. The authors showed that, after accounting for frequency scaling principles and the requirements of ISO 10140 and ISO 12999-1, the setup can provide reliable and repeatable results for sound insulation measurements of scaled specimens. However, the application of such setups to a systematic analysis of the influence of specimen dimensions and geometry on the measured value of the sound reduction index remains limited.

Many more studies can be found in the literature, in which the authors demonstrate and verify small-scale measurement setups used in sound insulation testing. Tsui et al. [26] presented small coupled reverberation rooms intended for transmission loss measurements of small panels, emphasizing the possibility of using such a solution as a low-cost setup for testing small specimens. Djambova et al. [27] proposed a small acoustic chamber for comparative measurements of the sound insulation of materials, indicating that such setups can be used as tools for assessing differences between materials under conditions simplified relative to full-scale laboratory tests. For small technical elements, such as air inlets, Puig et al. [28] also emphasized that the measured acoustic performance may be strongly affected by the coupling between the tested element and the acoustic rooms, particularly in the low-frequency range. Kling [29], in turn, analyzed the problem of scaling a wall test facility, pointing out that the miniaturization of building acoustics problems is more complex than in classical room acoustics because it requires considering not only the acoustic field in air, but also structural vibrations, transmission through elements, and boundary conditions.

Importantly, the aforementioned small-scale measurement setups can be applied to two distinct types of testing. First, they allow for the investigation of samples that are downscaled in accordance with established similarity criteria. Second, they can be utilized for testing certain full-scale samples, i.e., without scaling their thickness or material properties. In the latter case, however, the volume of the small reverberation chambers limits the feasibility of measurements in the low-frequency bands, proportionally to the original chamber dimensions and their scaling factor.

The aim of this study is to investigate how the dimensions and geometry of the test opening, and thus the effective dimensions of the specimen, affect the measured value of the sound reduction index *R*. The tests were carried out using small coupled reverberation rooms, however, even though small-scale setup were utilized, the tested samples retained their full-scale thicknesses and material properties, bearing in mind the limitations in terms of low-frequency measurement capabilities. Six different test openings were tested: three square openings, one quasi-square opening, and two rectangular openings. This configuration made it possible to compare specimens with different areas, as well as specimens with similar areas but different geometries. As a result, the influence of specimen area and specimen shape could be analyzed separately.

The study included materials with different mechanical and structural properties: steel, polymethyl methacrylate (PMMA), medium-density fiberboard (MDF), gypsum board, and Sylomer. These materials differ in thickness, density, stiffness, and internal structure, which makes it possible to assess whether the influence of specimen dimensions is universal or depends on the properties of the tested material. For each configuration, the measurements were repeated to evaluate not only differences in the mean values of the sound reduction index *R*, but also the repeatability of the obtained results.

The main contribution of this study is a systematic experimental analysis of the influence of specimen dimensions and geometry on the measured value of the sound reduction index for materials with different stiffness and structure, carried out using a controlled setup consisting of small coupled reverberation rooms. The obtained results may support the interpretation of sound insulation measurements performed on different-size specimens and the assessment of the suitability of small coupled reverberation rooms for comparative testing of non-standard materials and insulating elements.

## 2. Materials and Methods

To investigate the influence of specimen dimensions and test-opening geometry under controlled conditions, measurements were carried out in small coupled reverberation rooms using a set of material specimens installed in six different test openings. This section describes the measurement stand, the tested materials and specimen configurations, and the measurement procedure used to determine the sound reduction index *R*. These elements are presented in Section 2.1, Section 2.2, and Section 2.3, respectively.

### 2.1. Measurement Stand

**Figure 1:**
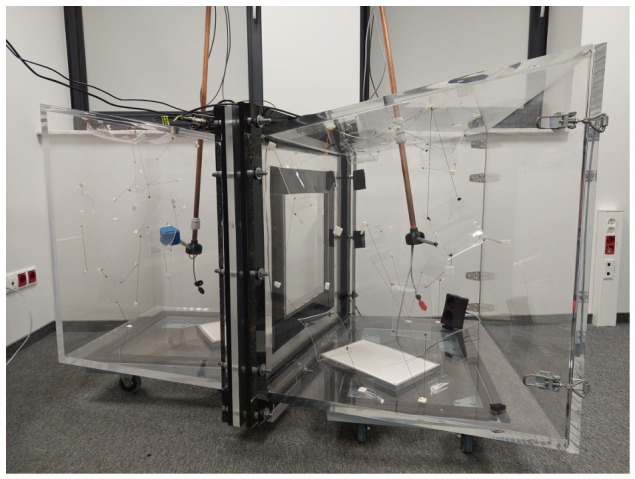
Measurement stand—small-scale coupled reverberation rooms.

The measurements were carried out using a dedicated small-scale coupled reverberation room stand, shown in Figure 1. The stand consists of a source room and a receiving room separated by a partition with interchangeable test openings. Its main components include microphones, loudspeakers, sound-diffusing elements, and sound-absorbing elements.

**Figure 2:**
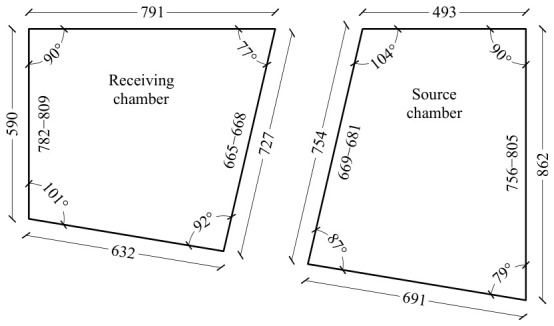
Measurement stand—small-scale coupled reverberation rooms: sketch with dimensions in mm. The intervals indicate the wall heights, which vary along the width Szeląg et al. [24].

The coupled reverberation rooms are a 1:8 scale model of the full-size reverberation rooms at the Department of Mechanics and Vibroacoustics of AGH University of Krakow. The source and receiving rooms have similar volumes of approximately $0.35\;m^{3}$. The room dimensions are shown in Figure 2. A detailed description and validation of the stand were presented by Szeląg et al. [24].

The study presented in this paper were conducted on full-scale, i.e., unscaled, samples, and the sound reduction index *R* was determined within the frequency range of 400Hz to 20kHz. This range was selected based on the dimensions of the used reverberation rooms and the standard requirements for test facilities. Specifically, according to ISO [5], reverberation rooms must have a minimum volume of $50\;m^{3}$, to enable sound insulation measurements starting from a frequency of 50Hz, while inherently taking into account certain methodological errors associated with an insufficiently diffuse acoustic field at low frequencies. Given that the tests were performed on full-scale samples without scaling their parameters, and furthermore, without scaling the measurement frequencies, the final measurement range narrows to a lower limiting frequency that is 8 times higher, i.e., 400Hz. It should be added that the original reverberation rooms are over 4 times larger than required by the standard—they have volumes of approximately $180\;m^{3}$ each; hence, the issues regarding acoustic field modality at low frequencies will be significantly less pronounced than would be expected for smaller rooms. In the high-frequency range, the test stand does not introduce significant limitations regarding the accuracy of the obtained measurement results.

### 2.2. Measurement Samples

The selection of measurement samples was intended to assess how the size of the test opening affects the sound reduction index *R* for materials with different mechanical and structural properties. Therefore, materials differing in stiffness, acoustic impedance, porosity, and microstructure were considered. During the selection process, parameters such as elastic modulus *E*, density ρ, thickness *t*, and surface mass $m^{\prime }$ were taken into account. Preliminary measurements were performed for seven materials, however, ultimately, five materials were selected for the final analysis: 1.0mm-thick steel, 3.8mm-thick PMMA, 3.2mm-thick MDF, 12.5mm-thick Sylomer (HD 100), and 12.5mm-thick gypsum board. Two materials tested during the preliminary stage were excluded from the final analysis. The first was 0.6mm-thick steel, for which the results were similar to those obtained for 1.0mm-thick steel, but shifted in frequency. The second was rubber, which was porous and permeable, resulting in very low and similar sound reduction index values for the tested configurations. The selected material parameters are summarized in Table 1. It is worth noting that the test samples were selected to correspond to some actual building materials if all their properties were scaled to the full dimensions of the reverberation chambers. However, this is of no consequence for the interpretation of the obtained measurement results. It was merely intended to assist in the proper design of the study, including the appropriate utilization of the measurement capabilities of the used experimental setup.

The measurement stand used for the tests was equipped with six different test openings: three square openings, one quasi-square opening, and two rectangular openings. The square openings measured 87.5×87.5mm, 175×175mm, and 350×350mm, while the quasi-square opening measured 615×675mm. The two rectangular openings measured 125×250mm and 250×500mm. The dimensions, surface areas, and opening-to-partition area ratios are summarized in Table 2. It should be noted that the 175×175mm square opening and the 125×250mm rectangular opening have nearly the same surface area but different geometries. A similar relationship exists for the 350×350mm square opening and the 250×500mm rectangular opening. This configuration allows the sound reduction index results to be compared for specimens with similar surface areas but different shapes.

**Figure 3:**
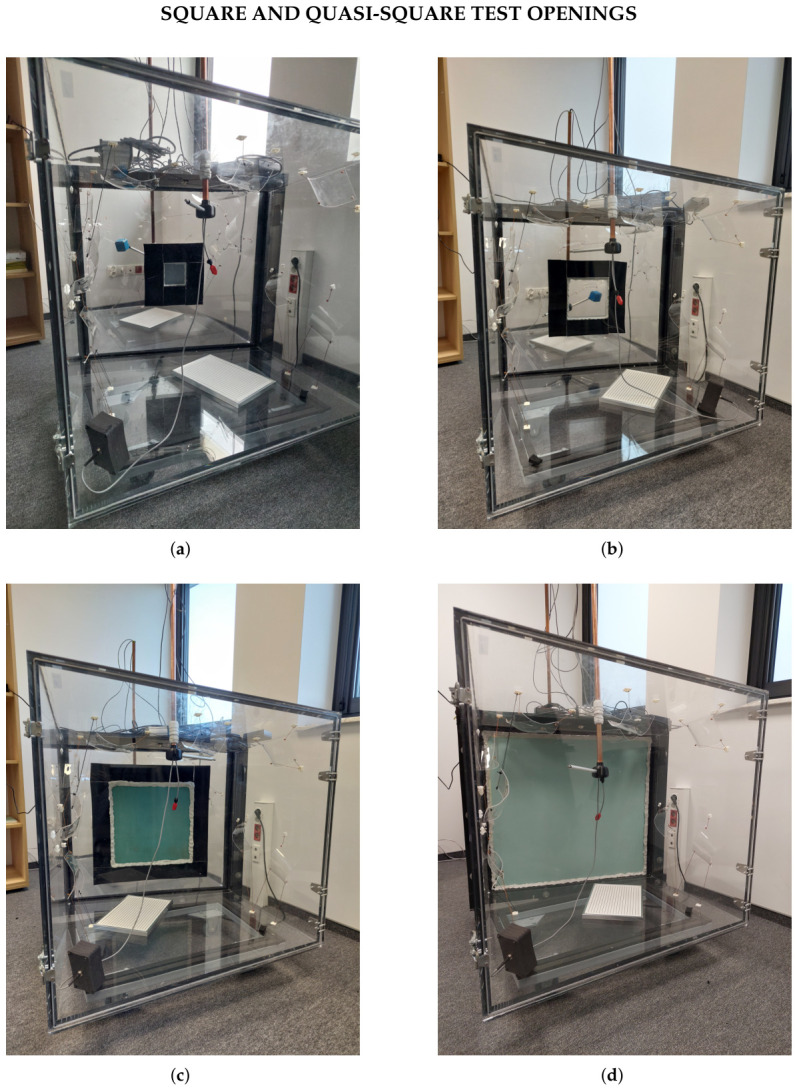
Photographs of specimens installed in four different square or quasi-square test openings: (**a**) steel, 87.5×87.5mm; (**b**) PMMA, 175×175mm; (**c**) Sylomer, 350×350mm; (**d**) gypsum board, 615×675mm.

**Figure 4:**
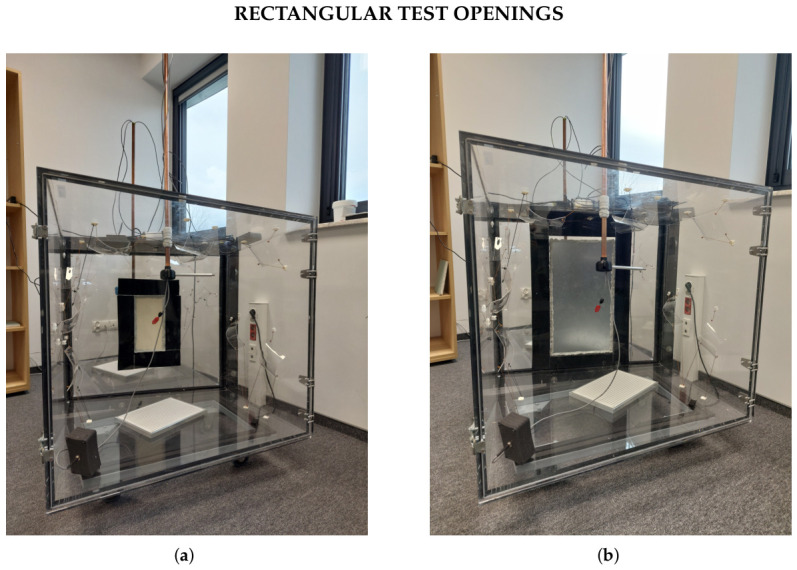
Photographs of specimens installed in two different rectangular test openings: (**a**) MDF, 125×250mm; (**b**) steel, 250×500mm.

The main objective of this part of the study was to determine how the size of the test opening affects the measured values of the sound reduction index *R*. Therefore, for almost all selected materials, specimens were prepared in sizes corresponding to all test openings. In the case of Sylomer, only the four smaller specimen sizes were used because of the limited availability of the material and its high compliance, which made proper sealing of larger specimens difficult. Table 3 summarizes the calculated fundamental resonance frequencies ($f_{11}$) for the investigated specimens for all five materials and for the considered specimen sizes and geometries. At the lowest investigated frequency (400Hz), the acoustic wavelength is $\lambda_{0}=0.86\;m$. Consequently, the largest and the smallest measurement openings correspond to approximately $0.79\lambda_{0}$ and $0.10\lambda_{0}$, respectively. Under these conditions, wave phenomena are expected to play a significant role in the measured sound insulation, contributing to the greater variability of the results and the higher standard deviations observed in the low-frequency range. Figure 3 and Figure 4 show the square and rectangular test openings used in the measurements, with example specimens installed in the stand. The specimens were made of five different materials selected to represent construction or industrial insulation elements with different mechanical and structural properties.

### 2.3. Measurement Procedure

The measurements were performed according to the procedure described in ISO 10140-2 and ISO 10140-4 ISO [2,4]. The sound reduction index *R* was determined from sound pressure level measurements in the source and receiving rooms. It was calculated using the energy-averaged sound pressure level in the source room, $L_{1}$ (dB), the energy-averaged sound pressure level in the receiving room, $L_{2}$ (dB), the area of the free test opening in which the specimen was installed, *S* ($m^{2}$), and the equivalent sound absorption area in the receiving room, *A* ($m^{2}$), according to Equation (1) ISO [4]:

(1) $$R=L_{1}-L_{2}+10\log_{10}\left(\frac{S}{A}\right).$$

The value of $L_{1}$ was obtained from ten sound pressure measurements in the source room: five microphone positions for the first loudspeaker position and five microphone positions for the second loudspeaker position. Similarly, $L_{2}$ was obtained from ten sound pressure measurements in the receiving room using the same measurement configuration. The reverberation time required for the determination of the equivalent sound absorption area A was determined from the room impulse response obtained using sweep-sine excitation and evaluated using the Schroeder backward integration method. To ensure the reliability of the results, each specimen was tested three times. Each measurement series was performed according to the ISO 10140 procedure, including independent reverberation time and background noise measurements. Between individual measurement series, the specimen was removed from the test opening, reinstalled, and carefully sealed. The final results were calculated as the arithmetic mean of the repeated measurements, and the standard deviation was calculated to assess measurement repeatability.

**Figure 5:**
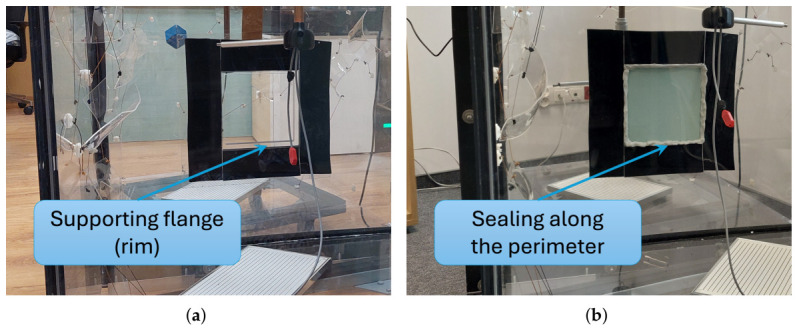
Specimen mounting and sealing in the test opening: (**a**) supporting flange (rim) of the test opening; (**b**) specimen pressed against the flange and sealed along the perimeter with non-setting putty.

The test opening was equipped with a supporting flange (rim) on which the specimen rested; therefore, the specimens were prepared with dimensions larger than the free test-opening dimensions. During installation, the specimen was pressed against the flange and sealed along the perimeter using a non-setting putty (sealing compound). This mounting condition corresponds to a resiliently supported (resiliently clamped) specimen (Figure 5).

## 3. Results

**Figure 6:**
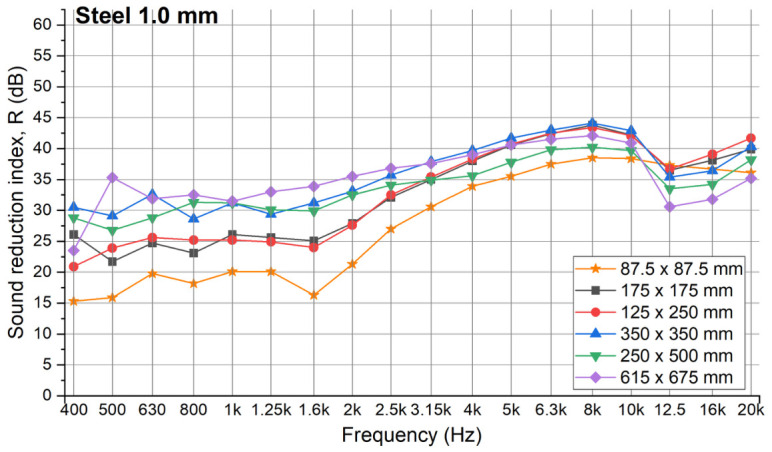
Sound reduction index *R* for 1.0mm-thick steel specimens measured for different test-opening sizes in the frequency range from 400Hz to 20kHz.

**Figure 7:**
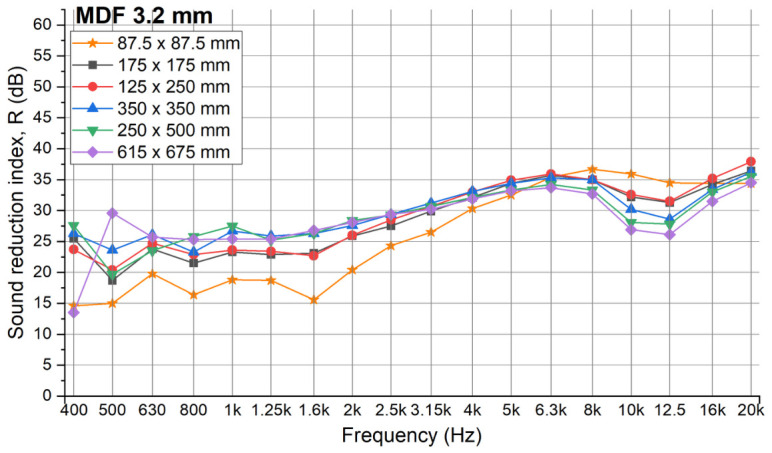
Sound reduction index *R* for 3.2mm-thick MDF specimens measured for different test-opening sizes in the frequency range from 400Hz to 20kHz.

**Figure 8:**
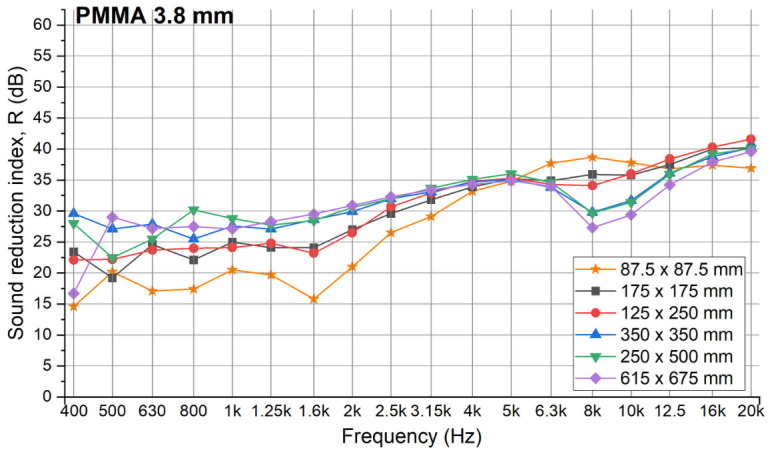
Sound reduction index *R* for 3.8mm-thick PMMA specimens measured for different test-opening sizes in the frequency range from 400Hz to 20kHz.

**Figure 9:**
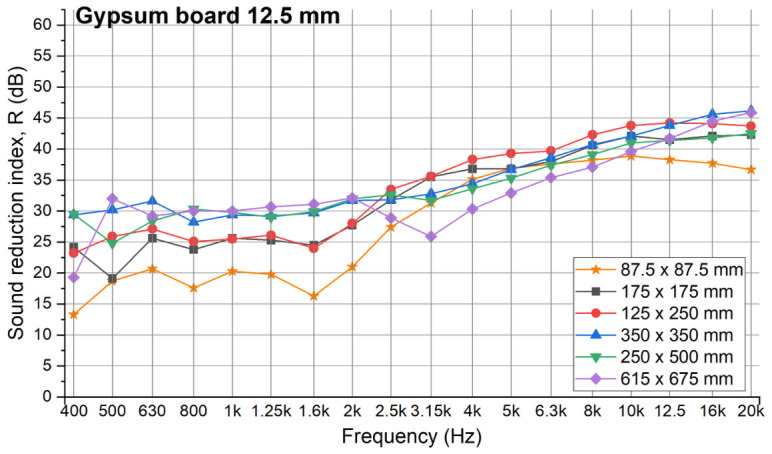
Sound reduction index *R* for 12.5mm-thick gypsum board specimens measured for different test-opening sizes in the frequency range from 400Hz to 20kHz.

**Figure 10:**
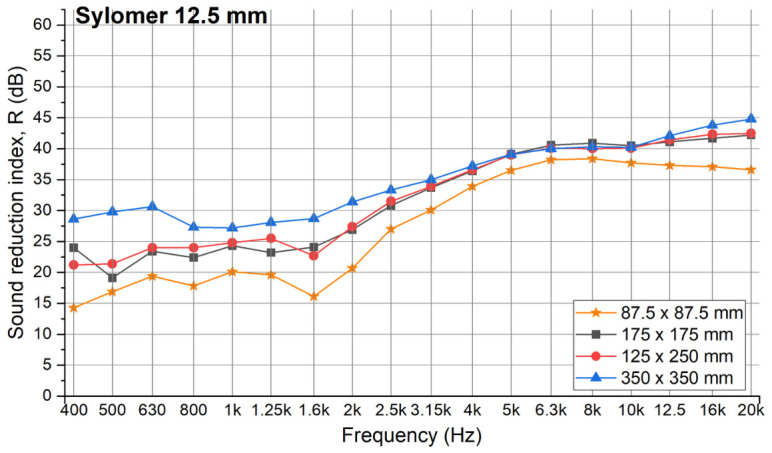
Sound reduction index *R* for 12.5mm-thick Sylomer (HD 100) specimens measured for different test-opening sizes in the frequency range from 400Hz to 20kHz.

The results of the sound reduction index *R* measurements are presented in Figure 6, Figure 7, Figure 8, Figure 9 and Figure 10. For clarity, each figure shows the mean values obtained for one material and for the subsequent test-opening sizes. The corresponding standard deviation values are presented in Table 4, Table 5, Table 6, Table 7 and Table 8. It is worth noting that it had been verified beforehand that all presented sound reduction index results maintain a margin of at least 10 dB from the upper measurement limit of the applied experimental setup.

For each material, the influence of the test-opening size, and thus of the effective specimen dimensions, was analyzed. Materials with different stiffness, thickness, density, and internal structure were selected to determine whether specimen dimensions affect all tested materials in the same way. The analysis considered frequency as the main independent variable, while the differences between the obtained curves were interpreted concerning the material properties and the geometry of the tested specimens.

### 3.1. Steel

Figure 6 presents the sound reduction index *R* measured for the 1.0mm-thick steel specimens installed in square and rectangular test openings of different dimensions.

For the smaller specimens, lower sound insulation values were observed in the low- and mid-frequency ranges. This effect was particularly visible below approximately 2kHz (resonance frequency region), and remained noticeable up to approximately 4kHz. Above the coincidence region, located around 12kHz for the tested steel specimen, higher sound insulation values were observed for smaller specimens.

The standard deviation values for the steel specimens are listed in Table 4. The repeatability of the results depended on the specimen size and geometry. The lowest standard deviations were obtained mainly in the frequency range from 1.6kHz to 20kHz, especially for the square specimens with side lengths of 175mm and 350mm, as well as for the rectangular specimen measuring 125×250mm. For the larger specimens, especially those measuring 250×500mm and 615×675mm, the measurements were less repeatable over a wider frequency range. In these cases, the standard deviation reached values of up to 5.3dB, which was associated with the greater difficulty of sealing large and relatively thin specimens in the test openings. In particular, for the largest steel specimens cut from rolled steel sheets, achieving a perfectly airtight and repeatable seal along the entire perimeter was practically challenging; therefore, these results were interpreted with appropriate caution.

### 3.2. MDF

Figure 7 shows the sound reduction index *R* measured for the 3.2mm-thick MDF specimens installed in the different test openings.

The results indicate that the sound insulation values obtained for the smallest test opening were lower at frequencies below approximately 2.5kHz. At higher frequencies, the differences between the curves became less pronounced. Above the coincidence region, located around 12.5kHz, higher values of the sound reduction index were observed for smaller specimens.

The standard deviation values for MDF are presented in Table 5. The lowest standard deviations were generally obtained in the frequency range from 1.6kHz to 20kHz. The most repeatable results were obtained for the square specimens with dimensions of 175×175mm and 350×350mm. In contrast, the larger specimens, especially those measuring 250×500mm and 615×675mm, showed lower repeatability over a broader frequency range.

### 3.3. PMMA

Figure 8 presents the sound reduction index *R* measured for the 3.8mm-thick PMMA specimens.

For this material, the smallest test opening produced lower sound insulation values below approximately 2.5kHz. Above this range, the differences between the results obtained for different specimen sizes decreased. Above the coincidence region, located around 8kHz, higher sound insulation values were observed for smaller specimens.

The standard deviation values for PMMA are listed in Table 6. The repeatability of the PMMA measurements was generally good in the frequency range from approximately 800Hz to 20kHz. The observed differences between the test-opening sizes confirm that the measured sound reduction index of relatively stiff and lightweight plates can be sensitive to specimen dimensions, especially in the frequency ranges in which modal behavior and coincidence-related effects are significant.

### 3.4. Gypsum Board

Figure 9 shows the sound reduction index *R* measured for the 12.5mm-thick gypsum board specimens.

The results indicate that the smallest test opening produced lower sound insulation values below approximately 2.5kHz. In the frequency range between approximately 2.5kHz and 12.5kHz, higher values of the sound reduction index were obtained for smaller specimens. This behavior suggests that, for this material, the influence of specimen dimensions is not limited only to the lowest frequency range, but may also affect the measured values in the mid-frequency range.

The standard deviation values for gypsum board are presented in Table 7. The repeatability analysis showed that the lowest standard deviations were generally obtained in the frequency range from approximately 800Hz to 20kHz. The higher standard deviations observed in the 400–800Hz range (for example for the 125×250mm specimen) are attributed to the fact that this band overlaps with its fundamental resonance frequency, which increases the sensitivity of the results to boundary conditions and mounting repeatability. Moreover, the results confirm that careful installation and sealing are particularly important when measuring relatively large specimens in small-scale test openings.

### 3.5. Sylomer

Figure 10 presents the sound reduction index *R* measured for the 12.5mm-thick Sylomer (HD 100) specimens. Due to the limited availability of the material and its high compliance, only the four smaller specimen sizes were tested.

For Sylomer, the sound insulation values obtained for the smallest test opening were lower below approximately 3.15kHz. Above this frequency, the results obtained for the different specimen sizes became more similar. This indicates that, for a highly compliant material, the influence of specimen size is most visible in the lower frequency range, whereas at higher frequencies the measured values tend to converge.

The standard deviation values for Sylomer are shown in Table 8. The repeatability of the measurements was generally best for square specimens in the frequency range from approximately 1kHz to 20kHz.

## 4. Discussion

The results obtained in this study indicate that the measured value of the sound reduction index *R* depends not only on the material properties of the specimen but also on the dimensions and geometry of the test opening. This effect was observed for all tested materials; however, for Sylomer the measurements were limited to the smaller test openings, and therefore the trends for the largest openings could not be assessed. Accordingly, the magnitude and frequency dependence of the effect should be interpreted with caution when contrasting stiff, plate-like materials with the highly compliant Sylomer specimen. The results therefore support the assumption formulated in the Introduction that measurements performed on reduced-size specimens cannot always be interpreted as directly equivalent to measurements performed on larger elements without considering finite-size effects, boundary conditions, and material-dependent dynamic behavior.

The most pronounced deviations were obtained for the smallest specimen, corresponding to the opening 87.5×87.5mm. For all materials analyzed, this configuration produced the lowest values of *R* in the low-frequency range. For stiff materials, such as steel, PMMA, MDF, and gypsum board, the differences relative to the other specimens were particularly evident below approximately 2–2.5kHz. For Sylomer, this effect persisted up to approximately 3.15kHz, after which the results for different specimen sizes began to converge. This means that the smallest specimen should not be treated as representative of larger elements without additional interpretation because its result is strongly related to finite-size effects, edge support conditions, and modal behavior. This observation is consistent with theoretical descriptions of sound transmission through finite plates, according to which the sound insulation of a plate depends not only on its surface mass, but also on bending stiffness, damping, radiation efficiency, and the way the specimen is supported at its edges Davy [12], Brunskog [32], Kim et al. [33].

The observed size-dependent behavior is also consistent with previous experimental studies showing that specimen area and construction parameters can affect measured sound transmission loss. Wareing et al. showed that changes in specimen dimensions can affect transmission loss results, particularly below the critical frequency Wareing et al. [11]. The present results show a similar trend: the influence of test-opening size was most pronounced in the frequency range in which modal effects and finite-size behavior are expected to be important. At the same time, the results indicate that the magnitude of this effect is material-dependent. Therefore, the size of the sample should not be treated as a purely geometric parameter, but rather as a parameter that interacts with the stiffness, thickness, density and damping of the tested material. To interpret the observed size effects, it is useful to relate the specimen dimensions to the corresponding acoustic wavelength. The acoustic wavelength is given by $\lambda_{a}=c/f$. When the specimen dimensions become comparable to $\lambda_{a}$, pronounced finite-size and modal effects are expected. In this context, Table 3 provides the calculated fundamental resonance frequencies $f_{11}$ for the considered specimen sizes and boundary conditions, indicating the frequency range in which the first structural modes may contribute to the measured sound reduction index. In addition, the coincidence frequency (Table 1) marks the transition region in which bending-wave radiation becomes efficient; therefore, the interaction between specimen dimensions and the coincidence region may further modify the measured trends, particularly for stiff plate-like materials.

For steel, PMMA, MDF and gypsum board, smaller specimens tended to show lower values of *R* at lower frequencies, while at higher frequencies the differences between the curves decreased or changed in character. In some cases, higher values of *R* were observed for smaller specimens above the coincidence region. This may result from changes in radiation efficiency and from the reduced ability of smaller finite plates to radiate sound effectively in selected frequency ranges. This behavior is consistent with the fact that acoustic radiation from finite plates differs from the idealized behavior of infinite plates and depends on the dimensions of the radiating surface Davy [12], Villot et al. [13]. These results confirm that measurements performed on small specimens require particular caution, especially when the aim is to extrapolate the results to larger elements.

Comparison of square and rectangular specimens with similar surface areas provides additional insight into the relative importance of specimen surface area and geometry. The 175×175mm square opening and the 125×250mm rectangular opening had nearly the same surface area, as did the 350×350mm square opening and the 250×500mm rectangular opening. In these pairs, the measured *R* curves were generally closer to each other than the results obtained for square specimens whose surface areas differed by a factor of four. This suggests that, within the investigated range of materials, specimen geometries, and measurement conditions, specimen surface area had a greater influence on the measured sound reduction index than specimen shape, while specimen geometry played a secondary role. Nevertheless, the curves were not always identical, which suggests that the aspect ratio and edge configuration can still influence modal distribution, vibration shapes, and effective boundary conditions. This observation is important for the design of small-scale or reduced-size tests because it shows that geometry-related effects may refine, but typically do not dominate, the surface-area effect.

The results obtained for Sylomer differed from those obtained for the stiffer plate-like materials. For this highly compliant material, the influence of specimen size was mainly visible below approximately 3.15kHz, whereas above this frequency the curves for different specimen sizes became more similar. This may be related to the low stiffness and high internal damping of the material, which reduce the influence of discrete plate modes and lead to a different transmission mechanism than in stiff plates. The Sylomer results therefore show that the effect of specimen dimensions is not universal, but depends strongly on the mechanical behavior of the tested material. This is particularly relevant for non-standard acoustic materials, including elastomeric, porous, layered, or metamaterial-based solutions, for which standard assumptions developed for conventional building partitions may not be directly applicable.

The repeatability analysis performed in the present study complements the previously published by Szeląg et al. [24] validation of the small coupled reverberation room stand and provides additional information on the repeatability of measurements for different specimen sizes, geometries, and materials. The standard deviation values were generally lower in the mid- and high-frequency ranges, whereas larger deviations were observed in selected low-frequency bands and for some larger specimens. This result is consistent with the known sensitivity of laboratory sound insulation measurements to source and receiver positions, room geometry, and boundary conditions Dijckmans and Vermeir [19], Dijckmans et al. [20]. In the present study, an additional factor was the repeated removal, reinstallation, and sealing of each specimen between measurement series. Therefore, the obtained standard deviation values include not only the variability of the acoustic field in the rooms, but also the practical repeatability of specimen installation.

The largest deviations were observed for selected configurations in which proper and repeatable sealing was more difficult. In addition to the wave-based finite-size effects that dominate at low frequencies (as discussed above), part of the observed scatter and large deviations can be attributed to mounting and sealing repeatability. This was particularly evident for larger or more compliant specimens, for which maintaining identical contact conditions along the specimen perimeter was more challenging. These observations agree with studies emphasizing the importance of installation and support conditions in laboratory sound insulation measurements, especially for thin plates and small specimens Wang et al. [18]. In practical terms, this means that the mounting procedure is a critical part of reduced-size sound insulation measurements and should be controlled as carefully as the acoustic measurement procedure itself.

Among the tested configurations, the 175×175mm square opening appears to be a promising compromise between specimen size, measurement repeatability, and sensitivity to differences between materials. This opening was small enough to limit material consumption and facilitate specimen preparation, while still providing repeatable results for several tested materials. Nevertheless, the selection of an optimal test-opening size should depend on the material type and on the frequency range of interest. For compliant or difficult-to-seal materials, smaller openings may be preferable, whereas for stiffer plate-like materials larger openings may be needed to better represent finite-plate behavior over a broader frequency range.

## 5. Conclusions

This study investigated how specimen dimensions and test-opening geometry influence the measured sound reduction index *R* in a small-scale coupled reverberation room stand, using five materials and six opening configurations.

The results confirm that specimen dimensions can significantly affect *R*, particularly at low frequencies, where finite-size and modal effects are most pronounced. The smallest opening, 87.5×87.5mm, should be treated as a limiting case of reduced-size testing because it produced the largest deviations and the lowest low-frequency *R* values for most materials. The magnitude and frequency dependence of the size effect were material-dependent, with trends for the compliant Sylomer differing from those of the stiffer plate-like materials. However, Sylomer was not tested in the largest openings; therefore, this material-dependence conclusion for the largest specimens requires further verification.

For openings with similar surface areas but different shapes, the results suggest that, within the investigated range of materials, specimen geometries, and measurement conditions, specimen surface area had a greater influence on the measured sound reduction index *R* than specimen shape. Square and rectangular specimens with comparable areas generally produced more similar *R* values than square specimens whose areas differed by a factor of four, indicating that changes in area tended to have a stronger effect than changes in shape. At the same time, the remaining differences between the square and rectangular results indicate that specimen geometry (aspect ratio and edge configuration) can still influence the outcome.

Repeatability depended strongly on mounting and sealing quality, especially for large specimens. For the largest steel specimens, the repeatability was additionally limited by practical difficulties in achieving a perfectly airtight and repeatable seal; therefore, these results should be interpreted with appropriate caution.

Overall, the results support the use of small coupled reverberation rooms for comparative testing and preliminary screening, but they also show that reduced-size measurements require careful interpretation and should not be treated as direct substitutes for full-scale standardized tests. The proposed methodology may be particularly useful for comparative studies of novel acoustic materials and prototype building elements when only reduced-size specimens are available during the early stages of material development. Because the measured *R* values were sensitive to specimen size and geometry, the trends reported here should not be generalized to other scales, opening configurations, or mounting conditions without additional verification. This caution is especially relevant for combined and non-standard material/structural solutions, where size- and boundary-related effects may interact with additional transmission mechanisms.

The main limitation of this study is that the results were obtained using one stand and a limited set of materials and specimen configurations. Although the investigated materials represent a broad range of structural properties, including metallic, polymeric, and composite panels, further validation using additional material types would be valuable to confirm the generality of the observed trends. Future work should extend the database to additional materials and systems and include direct comparisons between reduced-size and full-scale measurements under equivalent boundary conditions.

## Figures and Tables

**Table 1:** Summary of material types, thicknesses, and physical parameters of the five selected specimens.

Material Type	Thickness *t* (mm)	Density ρ ($\mathrm{kg}/m^{3}$)	Surface Mass $m^{\prime }$ ($\mathrm{kg}/m^{2}$)	Stiffness Elastic Modulus *E* (GPa) *	Coincidence Frequency (kHz)
**Steel**	1.0	7150	7.15	200–210	≈12.0
**MDF**	3.2	1391	4.45	2.5–4.0	≈12.5
**PMMA**	3.8	1069	4.06	2.0–3.3	≈8.0
**Gypsum board**	12.5	670	6.38	2.0–3.0	≈3.0
**Sylomer HD 100**	12.5	608	7.60	∼0.015–0.030	>30.0

* Elastic modulus ranges were taken from the literature [30].

**Table 2:** Summary of test opening sizes and calculated opening-to-partition area ratios. The total partition area was 510,003 ${\mathrm{mm}}^{2}$.

Test Opening	Dimensions (mm)	Surface Area (${\mathrm{mm}}^{2}$)	Opening-to-Partition Area Ratio
**Square**	87.5×87.5	7656	1.5%
**Square**	175×175	30,625	6.0%
**Square**	350×350	122,500	24.0%
**Quasi-square**	615×675	415,125	81.4%
**Rectangular**	125×250	31,250	6.1%
**Rectangular**	250×500	125,000	24.5%

**Table 3:** Calculated fundamental resonance frequencies ($f_{11}$) for the investigated specimens assuming free and clamped boundary conditions.

		Dimensions (mm)					
		**Resonance Frequencies $f_{11}$ (Hz)**					
**Material**	**Boundary condition**	87.5×87.5	175×175	350×350	615×675	125×250	250×500
**Steel**	free	667	167	42	12	218	51
	clamped	1257	314	79	23	450	105
**MDF**	free	621	155	39	12	203	48
	clamped	1171	293	73	22	419	97
**PMMA**	free	863	216	54	16	282	66
	clamped	1627	407	102	30	582	135
**Gypsum board**	free	2642	661	165	49	864	202
	clamped	4982	1245	311	92	1782	414
**Sylomer**	free	244	61	15	–	15	–
	clamped	460	115	29	–	29	–

The real boundary condition is expected to lie between the two limiting cases, i.e., $f_{11}^{\mathrm{free}}\leq f_{11,\mathrm{real}}\leq f_{11}^{\mathrm{clamped}}$.

**Table 4:** Standard deviation of repeated measurements for 1.0mm-thick steel specimens. Test-opening dimensions are expressed in mm, while column headings from 400 to 20k denote one-third-octave band center frequencies in Hz. Standard deviation values exceeding the Maximum Standard Deviation are shown in gray in the table.

Test Opening	400	500	630	800	1k	1.25k	1.6k	2k	2.5k	3.15k	4k	5k	6.3k	8k	10k	12.5k	16k	20k
87.5×87.5	2.4	1.7	1.5	0.7	1.3	0.7	0.6	0.3	0.5	0.6	0.8	1.1	1.1	0.6	0.3	0.6	0.8	0.4
175×175	2.4	1.4	1.1	0.4	2.0	0.7	0.1	0.3	0.3	0.3	0.5	0.5	0.4	0.4	0.2	0.5	0.4	0.4
125×250	0.9	2.5	1.3	0.5	1.2	1.4	0.2	0.4	0.2	0.1	0.5	0.5	0.6	0.6	0.3	0.6	0.8	0.8
350×350	0.5	2.6	2.0	0.8	0.6	0.7	0.5	0.1	0.3	0.2	0.6	0.7	0.6	0.4	0.4	0.2	0.4	0.9
250×500	1.2	2.8	0.7	1.3	0.5	1.1	0.2	0.5	2.2	2.8	3.4	4.2	5.0	5.3	4.3	1.4	1.7	2.3
615×675	2.5	2.5	1.1	0.8	1.1	0.7	0.7	0.7	0.6	0.4	0.6	1.8	3.3	4.3	2.7	0.5	2.6	1.2
Typical Standard Deviation *	0.6	0.6	0.6	0.6	0.6	0.6	0.6	0.6	0.6	0.6	0.6	0.6	0.6	0.6	0.6	0.6	0.6	0.6
Maximum Standard Deviation *	1.3	1.3	1.3	1.3	1.3	1.3	1.3	1.3	1.3	1.3	1.3	1.3	1.3	1.3	1.3	1.3	1.3	1.3

* Values determined or estimated based on ISO 12999-1 recommendation [31].

**Table 5:** Standard deviation of repeated measurements for 3.2mm-thick MDF specimens. Test-opening dimensions are expressed in mm, while column headings from 400 to 20k denote one-third-octave band center frequencies in Hz. Standard deviation values exceeding the Maximum Standard Deviation are shown in gray in the table.

Test Opening	400	500	630	800	1k	1.25k	1.6k	2k	2.5k	3.15k	4k	5k	6.3k	8k	10k	12.5k	16k	20k
87.5×87.5	1.1	1.3	1.9	1.2	1.4	0.4	0.7	0.7	0.5	0.8	0.5	1.0	0.3	0.4	0.5	0.6	0.3	0.4
175×175	1.9	1.4	0.9	0.4	0.3	0.3	0.7	0.4	0.3	0.6	0.3	0.2	0.5	0.7	1.0	0.4	0.6	0.6
125×250	1.9	0.6	1.0	0.5	0.6	0.2	0.9	0.7	0.1	0.5	0.3	0.5	0.5	0.5	0.4	0.6	0.9	1.7
350×350	2.0	0.5	1.0	0.6	0.8	1.0	0.4	0.3	0.3	0.3	0.2	0.1	0.2	0.1	0.2	0.3	0.4	0.4
250×500	1.5	2.7	2.7	0.3	0.2	0.6	1.1	0.9	0.7	0.7	1.4	1.4	1.1	0.6	0.5	0.5	0.7	1.2
615×675	3.1	2.7	0.9	0.4	1.0	1.0	0.5	0.4	0.7	1.2	1.8	2.5	2.5	1.8	0.7	0.2	0.6	0.7
Typical Standard Deviation *	0.6	0.6	0.6	0.6	0.6	0.6	0.6	0.6	0.6	0.6	0.6	0.6	0.6	0.6	0.6	0.6	0.6	0.6
Maximum Standard Deviation *	1.3	1.3	1.3	1.3	1.3	1.3	1.3	1.3	1.3	1.3	1.3	1.3	1.3	1.3	1.3	1.3	1.3	1.3

* Values determined or estimated based on ISO 12999-1 recommendation [31].

**Table 6:** Standard deviation of repeated measurements for 3.8mm-thick PMMA specimens. Test-opening dimensions are expressed in mm, while column headings from 400 to 20k denote one-third-octave band center frequencies in Hz. Standard deviation values exceeding the Maximum Standard Deviation are shown in gray in the table.

Test Opening	400	500	630	800	1k	1.25k	1.6k	2k	2.5k	3.15k	4k	5k	6.3k	8k	10k	12.5k	16k	20k
87.5×87.5	2.6	2.6	1.2	0.8	1.5	0.4	0.7	0.5	0.7	1.0	0.8	1.1	0.7	0.4	0.5	0.5	0.6	0.7
175×175	0.9	2.2	2.5	0.5	0.9	1.3	0.8	0.8	0.3	0.8	0.8	0.4	0.8	0.9	0.6	0.4	0.1	0.2
125×250	0.8	1.9	1.2	0.8	0.6	0.8	0.8	0.5	0.1	0.2	0.3	0.7	0.7	0.6	0.5	0.6	0.7	0.7
350×350	0.9	3.4	0.4	1.1	0.5	1.1	0.2	0.8	0.4	0.3	0.6	0.9	1.4	0.4	0.2	0.3	0.9	1.4
250×500	2.4	2.1	0.8	0.2	0.2	0.9	0.7	0.3	0.3	0.2	0.2	0.8	0.9	1.1	0.5	0.4	0.8	2.0
615×675	2.0	2.1	1.7	1.4	0.7	1.0	0.5	0.1	0.5	0.7	1.3	2.3	2.1	0.9	0.9	1.4	1.5	2.2
Typical Standard Deviation *	0.6	0.6	0.6	0.6	0.6	0.6	0.6	0.6	0.6	0.6	0.6	0.6	0.6	0.6	0.6	0.6	0.6	0.6
Maximum Standard Deviation *	1.3	1.3	1.3	1.3	1.3	1.3	1.3	1.3	1.3	1.3	1.3	1.3	1.3	1.3	1.3	1.3	1.3	1.3

* Values determined or estimated based on ISO 12999-1 recommendation [31].

**Table 7:** Standard deviation of repeated measurements for 12.5mm-thick gypsum board specimens. Test-opening dimensions are expressed in mm, while column headings from 400 to 20k denote one-third-octave band center frequencies in Hz. Standard deviation values exceeding the Maximum Standard Deviation are shown in gray in the table.

Test Opening	400	500	630	800	1k	1.25k	1.6k	2k	2.5k	3.15k	4k	5k	6.3k	8k	10k	12.5k	16k	20k
87.5×87.5	2.2	1.5	1.3	1.0	1.1	0.8	0.7	1.0	0.8	0.5	0.8	0.8	0.8	0.3	0.6	0.8	0.7	0.9
175×175	1.9	1.9	1.2	0.7	1.6	1.1	0.5	0.3	0.5	0.7	0.3	0.5	0.1	0.2	0.8	2.0	1.3	1.2
125×250	5.1	4.1	2.5	1.4	0.7	0.3	0.9	0.3	0.3	0.3	0.3	0.7	0.3	0.4	0.3	0.5	0.6	0.4
350×350	0.4	5.2	2.3	1.8	1.9	0.7	0.9	0.4	0.3	0.6	0.1	0.2	0.3	0.5	0.7	0.6	0.6	0.3
250×500	0.8	1.4	0.9	1.2	1.7	0.9	0.2	0.6	0.3	0.4	0.5	0.7	1.0	1.3	1.2	2.3	2.5	1.2
615×675	0.6	1.2	0.8	0.7	1.5	0.9	1.6	1.3	0.4	0.4	0.4	0.7	0.9	0.8	0.9	1.3	1.7	2.0
Typical Standard Deviation *	0.6	0.6	0.6	0.6	0.6	0.6	0.6	0.6	0.6	0.6	0.6	0.6	0.6	0.6	0.6	0.6	0.6	0.6
Maximum Standard Deviation *	1.3	1.3	1.3	1.3	1.3	1.3	1.3	1.3	1.3	1.3	1.3	1.3	1.3	1.3	1.3	1.3	1.3	1.3

* Values determined or estimated based on ISO 12999-1 recommendation [31].

**Table 8:** Standard deviation of repeated measurements for 12.5mm-thick Sylomer (HD 100) specimens. Test-opening dimensions are expressed in mm, while column headings from 400 to 20k denote one-third-octave band center frequencies in Hz. Standard deviation values exceeding the Maximum Standard Deviation are shown in gray in the table.

Test Opening	400	500	630	800	1k	1.25k	1.6k	2k	2.5k	3.15k	4k	5k	6.3k	8k	10k	12.5k	16k	20k
87.5×87.5	2.3	1.1	1.0	2.0	1.3	1.2	0.8	0.9	0.9	1.0	1.0	1.0	0.7	0.6	0.7	1.2	0.9	1.1
175×175	1.3	0.5	1.3	0.9	0.2	0.2	1.2	0.3	0.8	0.1	0.4	0.6	0.4	0.6	0.7	0.3	0.6	0.8
125×250	0.7	2.6	0.4	0.7	1.1	1.1	0.6	0.7	0.1	0.3	0.7	0.1	0.2	0.1	0.2	0.3	1.1	1.2
350×350	1.4	1.7	1.9	1.8	0.5	0.3	0.4	0.4	0.5	0.0	0.4	0.6	0.6	0.7	0.4	0.3	0.4	0.4
Typical Standard Deviation *	0.6	0.6	0.6	0.6	0.6	0.6	0.6	0.6	0.6	0.6	0.6	0.6	0.6	0.6	0.6	0.6	0.6	0.6
Maximum Standard Deviation *	1.3	1.3	1.3	1.3	1.3	1.3	1.3	1.3	1.3	1.3	1.3	1.3	1.3	1.3	1.3	1.3	1.3	1.3

* Values determined or estimated based on ISO 12999-1 recommendation [31].

## Data Availability

All data are included in paper.
